# Platelet extracellular vesicles enhance the proangiogenic potential of adipose-derived stem cells in vivo and in vitro

**DOI:** 10.1186/s13287-021-02561-w

**Published:** 2021-09-09

**Authors:** Yanan Tang, Jiayan Li, Weiyi Wang, Bingyi Chen, Jinxing Chen, Zekun Shen, Jiaxuan Hou, Yifan Mei, Shuang Liu, Liwei Zhang, Zongjin Li, Shaoying Lu

**Affiliations:** 1 Vascular Surgery Department, The First Affiliated Hospital of Xi′an Jiaotong University, 277 West Yanta Road, Xi′an, 710061 Shaanxi China; 2 Nankai University School of Medicine, 94 Weijin Road, Tianjin, 300071 China

**Keywords:** Adipose-derived stem cells, Platelet-derived extracellular vesicles, Angiogenesis, Ischaemic hindlimb

## Abstract

**Background:**

Adipose-derived mesenchymal stem cells (ADSC)-based therapy is an outstanding treatment strategy for ischaemic disease. However, the therapeutic efficacy of this strategy is not ideal due to the poor paracrine function and low survival rate of ADSCs in target regions. Platelet extracellular vesicles (PEVs) are nanoparticles derived from activated platelets that can participate in communication between cells. Accumulating evidence indicates that PEVs can regulate the biological functions of several cell lines. In the present study, we aimed to investigate whether PEVs can modulate the proangiogenic potential of ADSCs in vitro and in vivo.

**Methods:**

PEVs were identified using scanning electron microscope (SEM), flow cytometry (FCM) and nanoparticle tracking analysis (NTA). The CCK8 assay was performed to detect proliferation of cells. Transwell and wound healing assays were performed to verify migration capacity of cells. AnnexinV-FITC/PI apoptosis kit and live/dead assay were performed to assess ADSCs apoptosis under Cocl_2_-induced hypoxia condition. The underlying mechanisms by which PEVs affected ADSCs were explored using real time-PCR(RT-PCR) and Western blot. In addition, matrigel plug assays were conducted and mouse hindlimb ischaemic models were established to investigate the proangiogenic potential of PEV-treated ADSCs in vivo.

**Results:**

We demonstrated that ADSC could internalize PEVs, which lead to a series of biological reactions. In vitro, dose-dependent effects of PEVs on ADSC proliferation, migration and antiapoptotic capacity were observed. Western blotting results suggested that multiple proteins such as ERK, AKT, FAK, Src and PLCγ1 kinase may contribute to these changes. Furthermore, PEVs induced upregulation of several growth factors expression in ADSCs and amplified the proliferation, migration and tube formation of HUVECs induced by ADSC conditioned medium (CM). In in vivo experiments, compared with control ADSCs, the injection of PEV-treated ADSCs resulted in more vascularization in matrigel plugs, attenuated tissue degeneration and increased blood flow and capillary density in ischaemic hindlimb tissues.

**Conclusion:**

Our data demonstrated that PEVs could enhance the proangiogenic potential of ADSCs in mouse hindlimb ischaemia. The major mechanisms of this effect included the promotion of ADSC proliferation, migration, anti-apoptosis ability and paracrine secretion.

## Background

Stem cell therapy is a promising therapeutic strategy for patients with chronic limb-threatening ischaemia and has been extensively studied over the past 10 years [1]. The mechanism underlying the therapeutic effect of this strategy is the promotion of in vivo neovascularization [2, 3]. Among the variety of stem cell candidates, ADSCs show obvious advantages for clinical use, including ease isolation, relative abundance, stable self-renewal capacity, multipotent differentiative potential and low immunogenicity [4, 5]. Furthermore, many preclinical studies have confirmed that transplantation of ADSCs into an ischaemic model accelerates angiogenesis primarily through the paracrine function of proangiogenic and antiapoptotic factors rather than through the direct formation of new vessels via differentiation [6, 7].

However, ADSCs have poor cell retention and survival rate in target areas which significantly limit its optimal in vivo therapeutic efficacy [8, 9]. Ischaemic tissues create an unfavourable microenvironment with increased oxidative stress and decreased oxygen content, leading to the damage and death of transplanted cells and reducing the capacity of transplanted cells to form new vessels [10, 11]. Therefore, increasing the proliferation, migration and antiapoptotic capacity and promoting the paracrine and proangiogenic abilities of transplanted stem cells are crucial for ADSC-based therapy [12, 13].

Platelet extracellular vesicles are small membrane vesicles, which include exosomes (30–100 nm in diameter) and microvesicles (PMVs, 100–1000 nm in diameter), that are shed by activated platelets [14, 15]. Platelet extracellular vesicles are the most abundant cell-derived extracellular vesicles in circulation and exhibit multiple biological activities. PEVs can directly bind to cells and modify their functional properties. For example, PEVs can bind to leukocytes and haematopoietic stem/progenitor cells, activate leukocyte phagocytosis and enhance hematopoietic stem cell engraftment [16, 17]. Additionally, PEVs also act as intercellular carriers and affect recipient cell functions by delivering bioactive proteins, DNA and RNA to recipient cells [18]. It has been reported that PEVs receive plentiful proangiogenic growth factors and microRNAs, including VEGF, PDGF, FGF, microRNA223 and miroRNA34, from platelets. PEVs can transfer these molecules to endothelial cells and exert proangiogenic effects by promoting endothelial cell proliferation, migration and tubule-like structure formation and by protecting them from apoptosis [19–21].

The present study aimed to find out whether PEVs may promote the therapeutic effects of engrafted ADSCs against ischaemic injury and investigated the underlying molecular mechanisms.

## Methods

### Preparation and characterization of PEVs

Peripheral venous blood was collected from healthy volunteers by venipuncture with a 21-gauge needle and placed in an anticoagulant tube containing acid citrate dextrose (ACD).The blood samples were centrifuged at 150 g at room temperature for 25 min to remove the cells and obtain platelet-rich plasma (PRP). The PRP was centrifuged again at 750 g for 15 min to obtain the platelet sediment. After washing the pellet twice, the platelets were resuspended in sterile HEPES buffer and incubated with 1 U/ml thrombin for 90 min at 37 °C to produce PEVs. Following activation, the residual platelets were removed by centrifugation at 4 °C at 5000 g for 15 min. The supernatants containing the PEVs were harvested and pelleted for 90 min at 20,000 g at 4 °C. Finally, the pellets containing the PEVs were resuspended in PBS for use. The protein concentrations of PEVs were determined using BCA protein assay kit (Aksomics, China). All the volunteers signed informed consent forms, and the procedure was approved by the Ethics Committee of the First Affiliated Hospital of Xi'an Jiaotong University, Shaanxi, China.

The sizes, size distributions and structures of the PEVs were analysed using NTA and SEM. The phenotypes of the PEVs were assessed using flow cytometry (FCM). PEVs pellets were resuspended in 500 μl binding buffer; 100 μl PEVs were extracted and then incubated with FITC-conjugated Annexin V, PE-conjugated CD41a or the respective isotype control for 30 min. After centrifugation at 20000 g at 4 °C for 30 min, the pellets were resuspended in 500ul PBS for analysis using FCM.

### Isolation and identification of ADSCs

ADSCs were isolated from adipose tissue harvested from the abdomen of three females who underwent liposuction in the First Affiliated Hospital of Xi'an Jiaotong University, Shaanxi, China. The age of these three female donors ranged from 20 to 30 years, and preoperative examination proved they were all healthy. All of them provided informed consent, and the procedure was approved by the hospital Ethics Committee. After washing with phosphate-buffered saline (PBS), 10 ml adipose tissues were mechanically cut into 1-mm^3^ fragments and digested with 1% collagenase I and 1% trypsin for 90 min in a 37 °C water bath with intermittent shaking. The enzyme activity was terminated using Dulbecco’s modified Eagle’s medium (DMEM) (HyClone, USA) supplemented with 10% foetal bovine/bovine serum (FBS) (Gibco, USA). The suspension was centrifuged at 1200 rpm for 10 min at room temperature to remove the mature adipocytes and obtain a cell pellet. Subsequently, the cell pellet was resuspended and incubated overnight in complete DMEM-F12 medium. FCM analysis was used to characterize the phenotypes of the ADSCs. A total of 5 × 10^5^ cells were resuspended in 500 µl PBS and then incubated with fluorescence-labelled monoclonal antibodies against CD29, CD44, CD90, CD105, CD45 or CD34 (BD, USA) or the respective isotype control for 15 min. After washing, the labelled cells were analysed with FCM by using a fluorescence-activated cell sorter (FACS) (BD, USA).

### ADSC proliferation, migration and apoptosis assays

ADSCs were sowed at 2 × 10^3^/well with 100 µl medium in 96-well plates and then treated with PBS and different concentrations of PEVs (5, 10 or 20 µg/ml) for 24 h, 48 h and 72 h. After incubation, medium was removed and CCK-8 solution (10 µl of reagent in 100 µl culture medium) was added to each well, followed by colour development for 100 min. The OD value was measured at 450 nm using a microplate reader (Thermo, USA).

The capacity of the ADSCs to migrate was detected using transwell chambers (8.0 µm pore size, 24-well, Corning, USA). Then, 600 µl of DMEM-F12 containing FBS was used to promote cell migration to the lower chamber. A total of 2 × 10^4^ cells in serum-free medium (100 µl) containing PBS or different concentrations of PEVs were sowed in the upper chamber. After incubation at 37 °C for 15 h, the cells on the surface of the membrane were washed with PBS, fixed with paraformaldehyde for 20 min and stained with 0.5% crystal violet for 20 min at room temperature. ADSC cells on the top surface of the transwell membrane were scraped out by using a cotton swab. After that, five fields of each transwell were randomly selected to count the number of migrated cells on the back surface.

ADSCs (5 × 10^3^) were seeded in 96-well plates. The medium of the ADSCs treated for 24 h with PEVs was replaced with DMEM/F12 without FBS and supplemented with 700 ng/ml cobalt chloride. After 24 h of incubation, the normal, apoptotic and necrotic cells were stained with Calcein-AM and PI solution, respectively, followed by imaged under a fluorescence microscope (Leica, German). The number of live and dead cells was measured using ImageJ software. After seeding 5 × 10^4^ ADSCs in 6-well plates, PBS and different concentration PEVs were used to treated the cells for 24 h. Cocl2 was used to induce hypoxia condition as above mentioned. The apoptosis rate was detected using Annexin V-FITC/PI kit (Yeason, China) in accordance with the manufacturer’s protocols.

### Confocal laser scanning microscopy

ADSCs were transfected with eGFP gene recombinant virus, incubated with CM-Dil-labelled PEVs at the indicated concentrations for the indicated time intervals and analysed after fixation. Confocal imaging was performed with a confocal microscope.

### RNA isolation and real-time qPCR

ADSCs were cultured in basal medium supplemented with different concentrations of PEVs (5, 10 and 20 µg/ml) for 24 h, and PBS was utilized as the control treatment. Total RNA was extracted from ADSCs treated with various conditions using RNAiso Plus (Takara), according to the manufacturer’s protocols. The isolated RNA was subsequently reverse transcribed into cDNA with a PrimeScript™ RT Reagent Kit (Takara) with gDNA Eraser. The reaction mixture for qPCR containing SYBR Premix Ex Taq II (Takara, Japan) was prepared according to the manufacturer’s protocols. RT-PCR was performed in a PCR system with HGF, VEGF, FGF2, ANG1, ANG2, PGF, TGFβ and GAPDH primers. Relative gene expression was calculated using the 2 − ΔΔCT method, using GAPDH mRNA expression as reference gene. Each sample was analysed at least three times.

### Western blot assay

Protein samples were extracted from pretreated ADSCs and quantified using a BCA protein assay (Beyotime Biotechnology, China). After being separated on 10% SDS-PAGE gel, the protein samples were transferred to PVDF membranes (Millipore, USA). And after it is blocking with TBST containing 5% BSA and incubated with primary antibodies at 4 °C overnight. The following day, the membranes were then incubated with secondary antibodies, washed with TBST and visualized using a ECL system. Densitometric analysis was performed using ImageJ software, and relative protein expression was represented by the ratio of grey value of target band to GAPDH.

### Preparation of conditioned medium from ADSCs (ADSC-CM)

ADSCs were cultured in medium containing 10% FBS with PBS (control group) or different concentrations of PEVs (5, 10 or 20 µg/ml). When the cells reached 90% confluence, the culture medium was replaced with serum-free fresh DMEM-F12. Following 48 h of culture, the conditioned medium (CM) from different groups was collected and centrifuged at 800 rpm for 5 min to remove the suspended cells. The ADSC-CMs were stored in a − 80 °C freezer for subsequent experiments.

### HUVEC proliferation, migration and wound healing assays

HUVECs were purchased from American Type Culture Collection (ATCC, USA), and 2 × 10^3^/well cells were seeded in 96-well plates. After cultured with 100 µl FBS-free medium for 12 h, cells were then treated with ADSC-CMs from different group for 24 h, 48 h and 72 h. CCk8 assay was performed to detect the relative cell viability. And migration capacities of HUVECs cultured in different ADSC-CMs were evaluated using a Trans well assay. The detailed protocols were the same as those for ADSCs described above.

A wound healing assay was used to assess the migration of HUVECs as well. Scratches were created using 200-µl sterile pipette tips in six-well culture plates when the cells had grown to 100% confluence. After scratching, the cells were washed with PBS and cultured in ADSC-CMs. Photographs were taken 0 h and 24 h post-culture using an optical inverted microscope (Nikon, Japan).

### In vitro tube formation assay

The capacity of ADSC-CMs to stimulate angiogenesis was assessed with a tube formation assay. After thawing on ice at 4 °C overnight, 60 µl Matrigel (BD, USA) was added to each well of 96-well plates and allowed to solidify at 37 °C for 60 min in an incubator. HUVECs (1 × 10^4^) were seeded in each Matrigel-coated well and incubated with different types of ADSC-CM (100 µl) at 37 °C in 5% CO_2_ for 6 h. The tubular structures were observed under an inverted microscope. Tube length and network structural complexity (number of branches, number of junctions, number of meshes and tube length) were determined using angiogenesis analyser that is developed for the ImageJ software [22].

### In vivo Matrigel plug assay

A Matrigel plug assay was performed to evaluate the vasculogenic potential of the cells. PBS (50 µl), ADSCs (50 µl, 5 × 10^5^ cells) or P-ADSCs (48 h pretreatment with 20 µg/ml PEVs, 5 × 10^5^ cells) were mixed with 400 µl Matrigel, and the mixture was subcutaneously injected into the abdomens of 8-week-old male C57BL/6 mice. The Matrigel implants were harvested for immunohistochemistry (IHC) after 2 weeks. The animal experiments were conducted according to the guidelines and ethical standards of the Animal Care and Use Ethics Committees of Xi’an Jiao Tong University.

### Mouse ischaemic hindlimb model and ADSC transplantation

Hindlimb ischaemia was induced in 8-week-old wild-type C57BL/6 mice. After anaesthetization with isoflurane, the animals were placed on a warm pad in a supine position, and the hindlimb hair was removed with depilatory cream. The proximal and distal portions of the left femoral artery and its branches were ligated with double knots using 7–0 absorbable ligature, and the skin incision was closed using 5–0 silk sutures. After 24 h, pretreated ADSCs were resuspended in PBS, and 3 × 10^5^ cells were intramuscularly injected in 3 different sites of the ischaemic hindlimb (gastrocnemius, gracilis and quadriceps muscles, 30 µl per injection). A laser Doppler perfusion imager (MoorFLPI; Moor instruments, UK) was used to measure the blood flow recovery of the hindlimbs on days 0, 7, 14 and 21 after ADSC transplantation. The perfusion ratios of the ischaemic limb to the lateral nonischaemic limb were analysed in the three groups.

### Histological examination, immunohistochemistry and immunofluorescence

Three weeks after cellular transplantation, the mice were killed, and the hindlimb gastrocnemius muscle was removed and fixed with 4% formaldehyde at 4 °C for 24 h. After embedding in paraffin and sectioning, the samples were transversely sectioned into 5-µm-thick sections and stained with haematoxylin and eosin (H&E). To measure vascular formation in vivo, IHC was performed using CD31 (Abcam, UK) antibodies. The number of vessels was counted in three areas with the highest neovascularization. The average count of three fields was determined in each sample.

### Statistical analysis

The experiments were repeated three times. The quantitative results are expressed as the mean ± SEM. Student’s t test and ANOVA were used to analyse the variance between different groups. Values of P < 0.05 were considered statistically significant.

## Results

### Characterization and interaction of ADSCs and PEVs

**Fig. 1:**
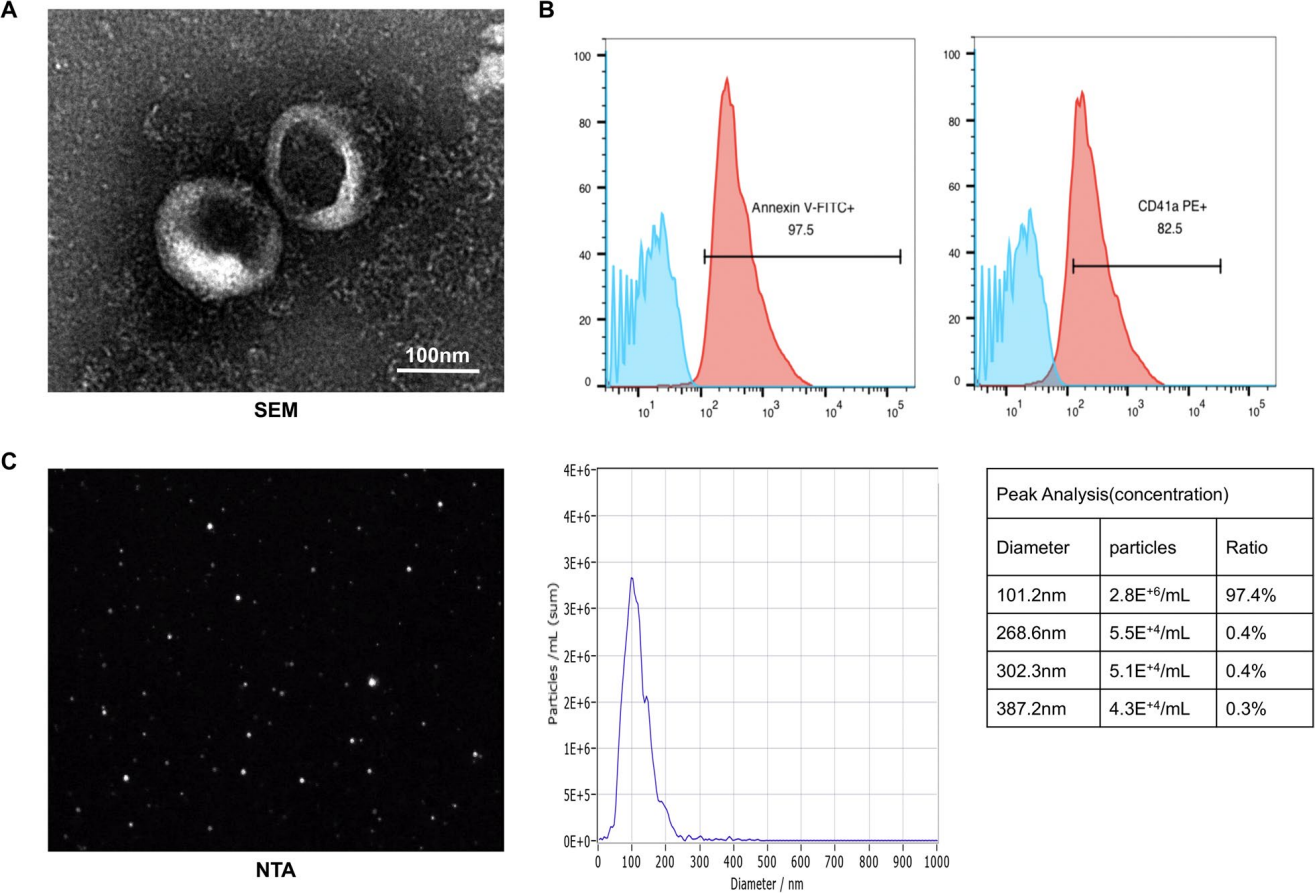
Identification of PEVs. **A** SEM showed that PEVs are spherical nanoparticles. **B**. FACS proved that Annexin V and specific markers CD41a  were highly expressed on PEVs surface. **C** Representative image and analysis data of NTA;

SEM and NTA proved that the PEVs were nanospherical particles with an average diameter of 100 nm (Fig. 1A, C). Furthermore, the flow cytometry results showed that the PEVs we extracted were strongly positive for Annexin V and the PLT-specific surface marker CD41a [23] (Fig. 1B).

**Fig. 2:**
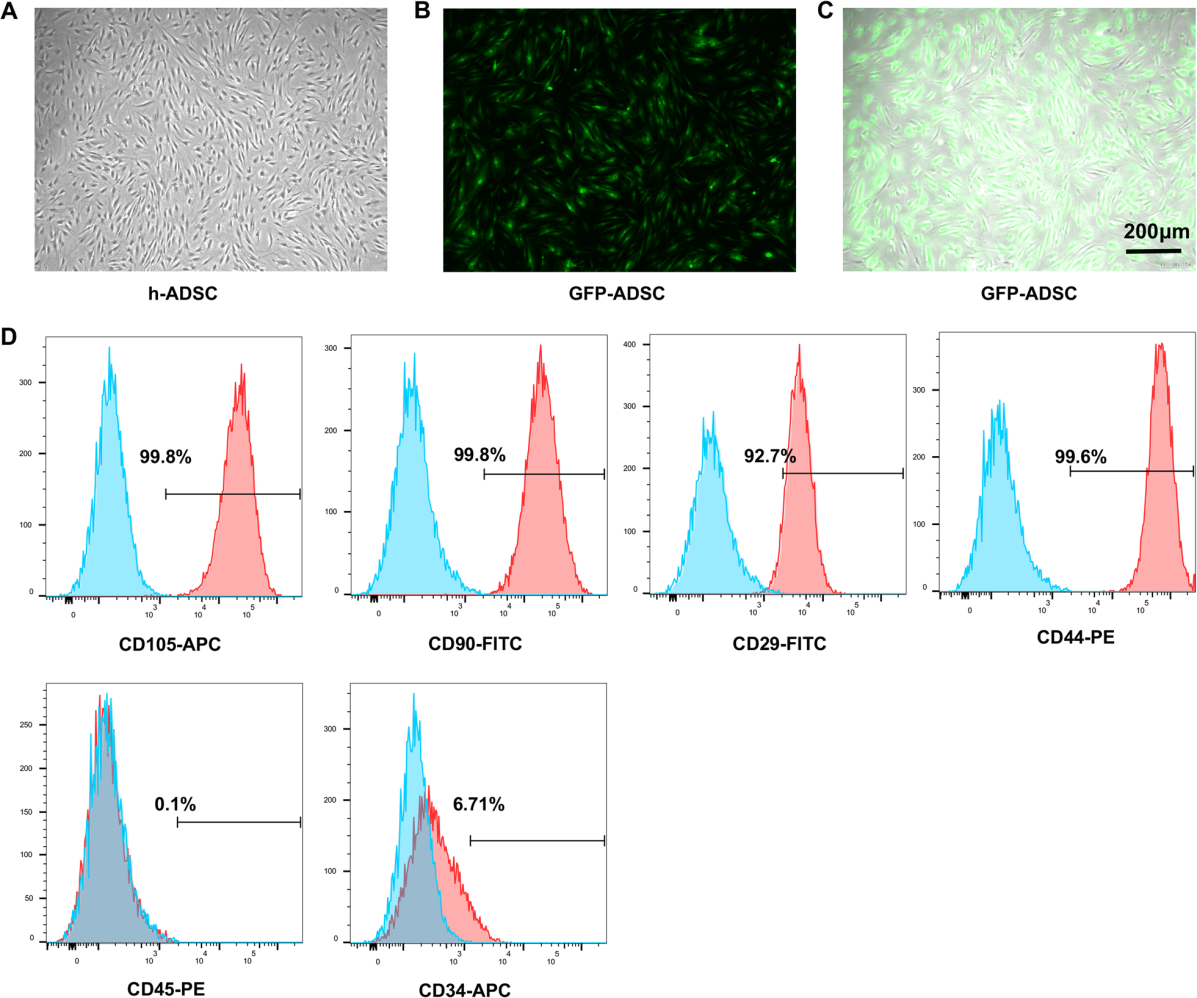
Identification of ADSCs. **A**. Representative image of spindle-shaped ADSC in passage 3. **B** Image of ADSC transfected with eGFP lentivirus in fluorescent field. **C** Merge image of ADSC transfected with eGFP lentivirus in fluorescent field and bright field. **D** FACS showed the percentage of CD29, CD44, CD105, CD90, CD34 and CD45 expression in the surface of ADSCs

As observed with an inverted microscope, ADSCs were spindle shaped and presented a vortex distribution when they reached 90% confluence (Fig. 2A). Isolated ADSCs were labelled with different cell surface markers to determine the cell phenotypes using FCM. The results indicated that the ADSCs were strongly positive for the stem cell surface markers CD29 (92.7%), CD44 (99.6%), CD90 (99.8%) and CD105 (99.8%) but did not express CD45 (0.1%) or CD34 (6.71%) (Fig. 2D).

**Fig. 3:**
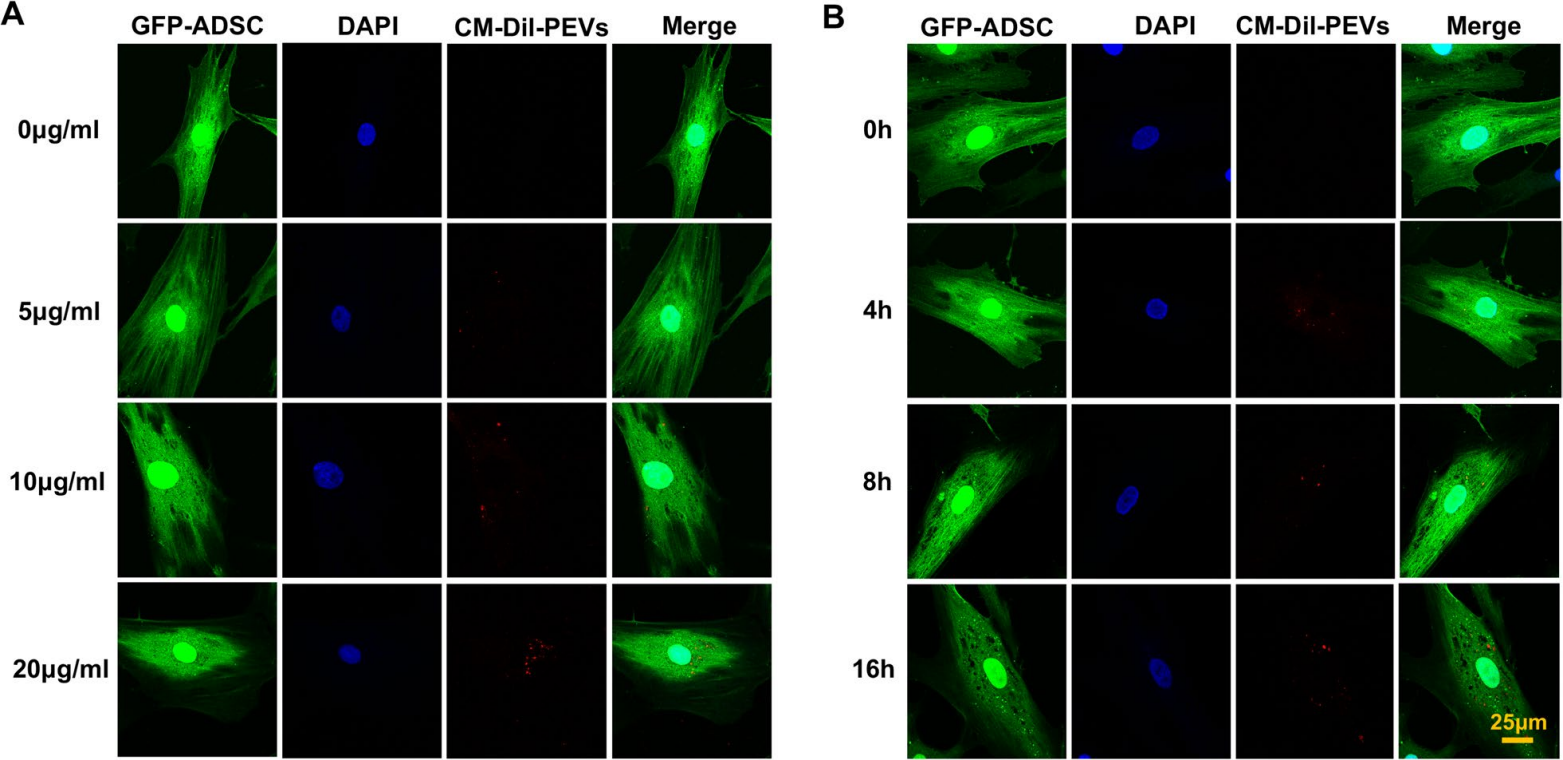
Interaction of PEVs with ADSCs. **A** Confocal laser scanning microscopy of eGFP-ADSC reacted with CM-Dil-labelled PEVs of different concentrations; **B** confocal laser scanning microscopy of eGFG-ADSC reacted with CM-Dil-labelled PEV at different time points

To characterize the interaction between the PEVs and ADSCs, we labelled the PEVs with CM-dil and transfected e-GFP into the ADSCs (Fig. 2B, C). Confocal laser scanning microscopy revealed that the CM-Dil-labelled PEVs could be internalized by the GFP-ADSCs. The internalization of PEVs was concentration and time dependent (Fig. 3A, B).

### PEVs promote ADSC proliferation, migration and anti-apoptotic capacities

**Fig. 4:**
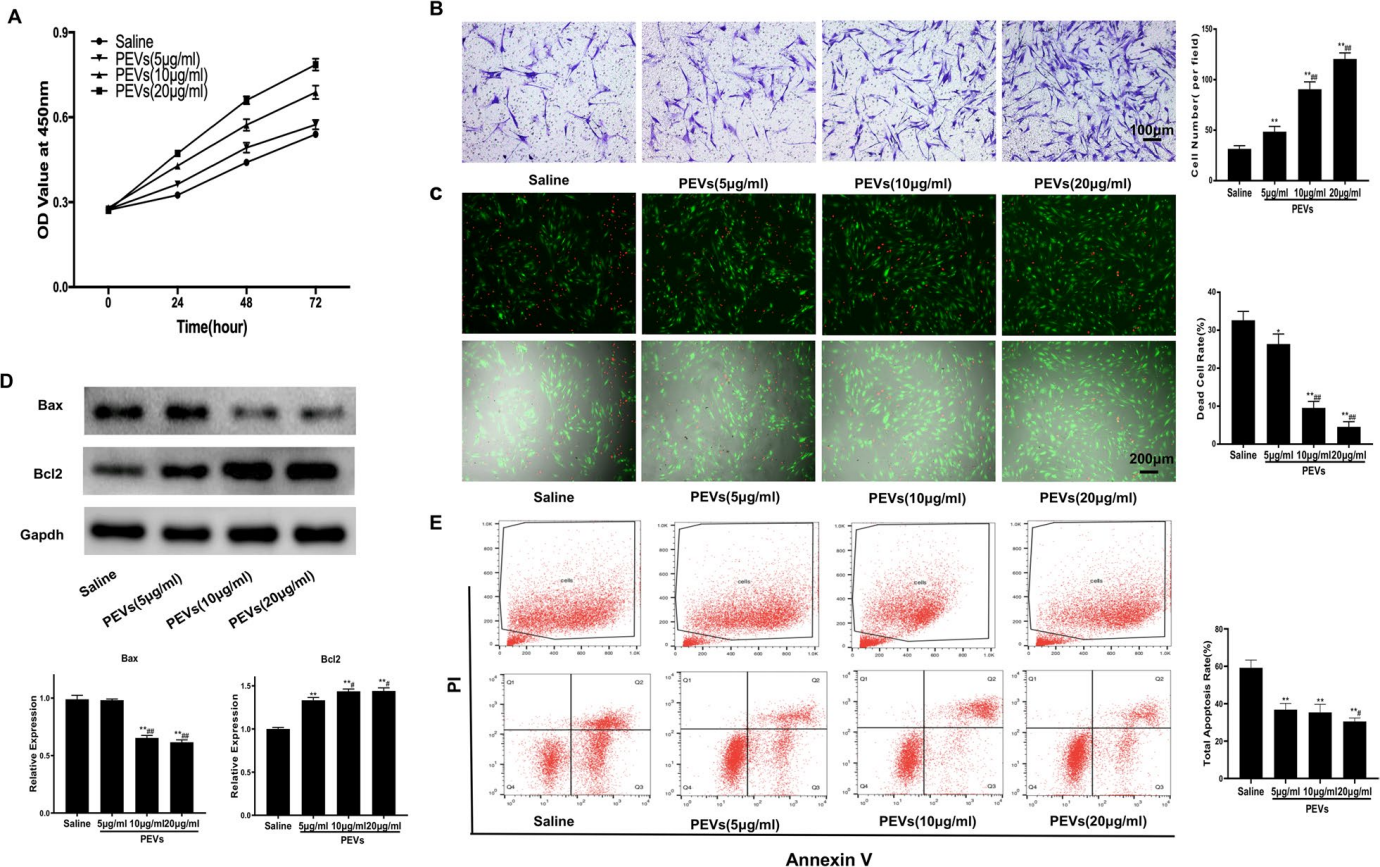
PEV preconditioning enhanced viability, migration and attenuated Cocl2-induced hypoxic apoptosis of ADSCs in vitro. After 24 h PEV pre-treatment, Cocl2(700 ng/ml) was used to induce hypoxic apoptosis of ADSCs. Cell apoptosis was evaluated by Live/dead staining and FACS. **A** Proliferation of ADSCs preconditioned with different concentration of PEVs for different periods of time. **B** Representative images and quantification of transwell assay. **C** Calcein-AM/PI staining showed that the dead cells were significantly reduced in the PEV preconditioning groups. The rate of dead cells was expressed as the ratio of dead cells to total cell count. **D** The apoptosis-related proteins Bax and Bcl2 were evaluated using Western blotting. **E** FACS showed that Cocl2-induced hypoxic apoptosis rate of ADSC was decreased in PEV preconditioning groups. Data are presented as the mean ± SEM. *p < 0.05, **p < 0.01 versus saline; #p < 0.05, ##p < 0.01 versus PEVs (5 µg/ml)

The CCK-8 assay data showed that the cell number was significantly increased in the PEV groups compared with the control group at 24 h and 48 h. Furthermore, 20 µg/ml PEVs exerted a stronger effect on ADSC proliferation than 5 µg/ml and 10 µg/ml PEVs (Fig. 4A). The results suggested that PEVs had the highest impact on ADSC proliferation when added to the culture medium at a concentration of 20 µg/ml for 48 h.

Cell migration from the transplant site to the ischaemic area is a vital step in stem cell-based therapy, so we detected the effect of PEVs on ADSC migration using a transwell chamber migration assay. The results indicated that more cells migrated through the transwell membrane in the PEV groups than in the PBS groups. Furthermore, ADSCs exhibited dose-dependent alterations in the PEV groups, and ADSCs showed better mobility when cultured with 20 µg/ml PEVs than when cultured with 5 µg/ml or 10 µg/ml PEVs (Fig. 4B). In summary, PEVs significantly promoted the migration of ADSCs.

The survival of ADSCs treated with or without PEVs under Cocl2-induced hypoxia condition was investigated by using calcein-AM/PI staining, Annexin V-FITC/PI apoptosis kit and Western blotting. The results showed that ADSCs treated with PEVs exhibited a lower rate of apoptosis and dead cells than ADSCs treated with PBS (Fig. 4C, E). We also observed higher expression of antiapoptotic protein bcl-2 and lower expression of apoptotic protein Bax in the ADSCs treated with PEVs compared with the ADSCs treated with PBS group (Fig. 4D).

### PEVs upregulate angiogenic gene expression in ADSCs

**Fig. 5:**
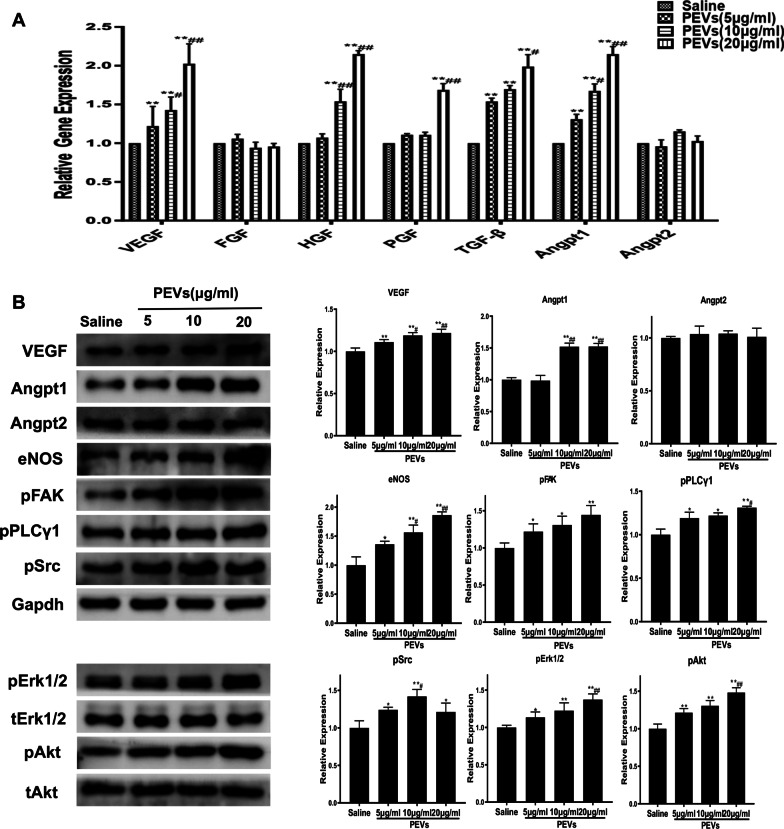
RT-PCR and Western blot showed the expression of various angiogenic-related genes in P-ADSC were upregulated. **A** The mRNA level of VEGF, FGF, HGF, PGF, TGF-β, Angpt1,Angpt2 in all groups were assessed using RT-PCR; **B **Protein levels of VEGF, Angpt1, Angpt2, eNOS, pAkt, pErk1/2, pFAK, pSrc and pPLCγ1 were assessed using Western blot. Data are presented as the mean ± SEM. *p < 0.05, **p < 0.01 versus Saline; #p < 0.05, ##p < 0.01 versus PEVs (5 µg/ml)

To investigate the effect of PEVs on ADSC proangiogenic ability, as well as the potential underlying mechanism, we quantified the expression levels of angiogenic-related genes and pathways by using RT-PCR and Western blotting. As shown in Fig. 5, we observed that VEGF, HGF, PGF, TGF-β and Angpt1 expression markedly increased in the PEV-treated ADSCs compared with the control-treated ADSCs. Moreover, the VEGF, HGF and Angpt1 mRNA levels in the PEV groups were correlated with PEV concentration. The mRNA expression of these genes showed an trend of increasing with increasing PEV concentration (Fig. 5A).

The protein levels of VEGF, ANG1, ANG2, eNOS, pAkt and pErk in all the cell groups were measured using Western blotting. Compared with the control group, all the PEV concentration groups showed higher expression of VEGF, ANG1, eNOS, pAKT and pErk. There were no differences in ANG2 expression among the groups (Fig. 5B).

### PEVs improve the effects of ADSC-CM on HUVEC viability, migration and tube formation

**Fig. 6:**
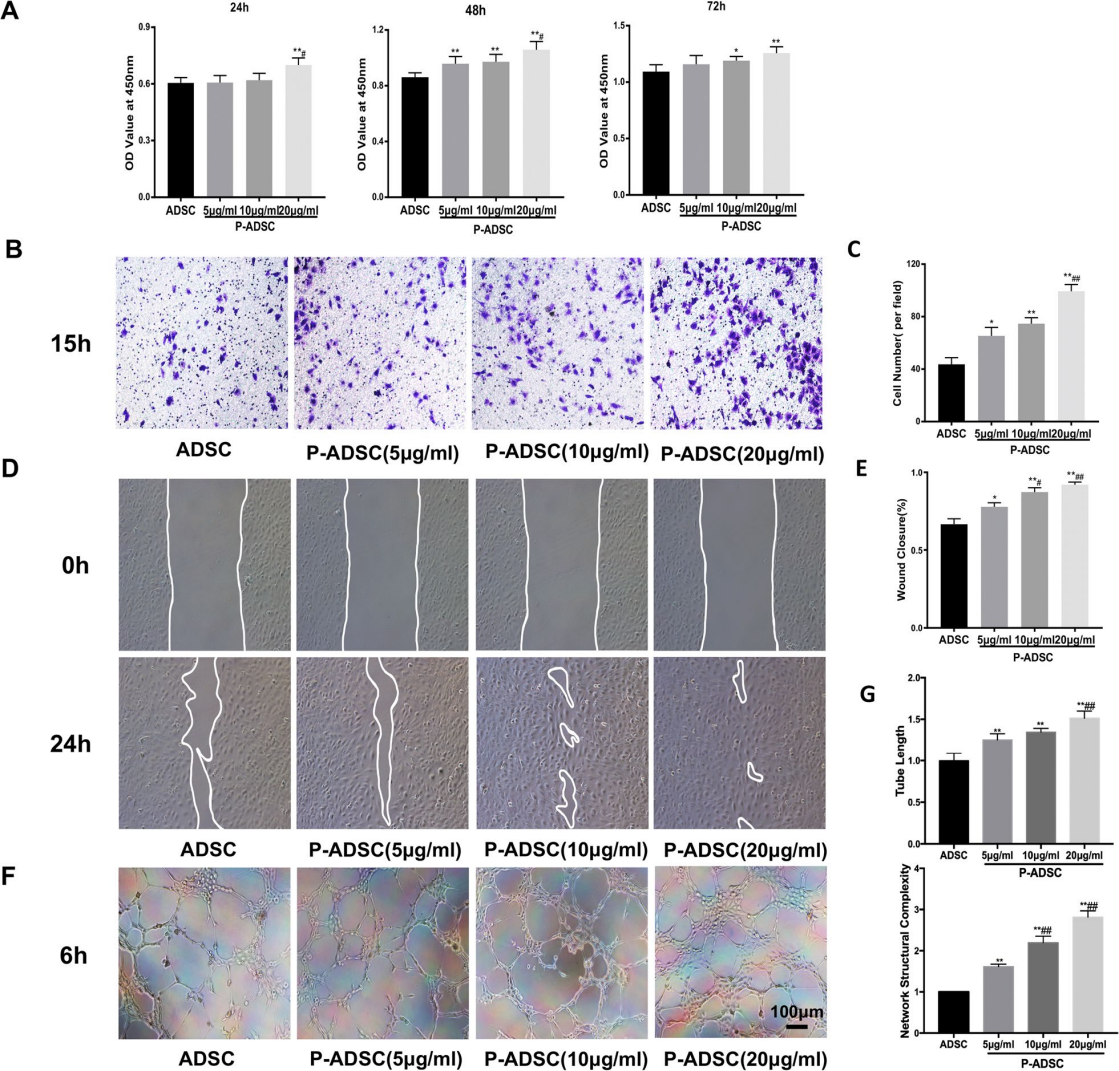
Cultured-medium derived from P-ADSCs promoted the proliferation, migration and tube formation of HUVECs. **A** Cell proliferation was measured using CCK8 assay; **B**, **C** representative images and analysis of HUVEC migration treated with CM from ADSC and P-ADSC; **D**, **E** representative images and analysis of HUVEC wound healing assay; **F**, **G** tube formation assay images and quantification of tube length and network structural complexity. Data are presented as the mean ± SEM. *p < 0.05, **p < 0.01 versus saline; #p < 0.05, ##p < 0.01 versus PEVs (5 µg/ml)

First, we detected the effect of ADSC-CM from different groups on HUVEC viability using a CCK-8 assay. The results suggested that the OD value of the HUVECs cultured in ADSC-CM from the PEV group was significantly higher than that of the HUVECs cultured in ADSC-CM from the PBS group. In addition, we found that when ADSCs were pretreated with 20 µg/ml PEVs, the conditioned medium showed the strongest ability to promote endothelial cell proliferation (Fig. 6A). Then, we measured the migration capacity of HUVECs cultured in the AVSC-CMs via transwell and wound healing assays. We observed that ADSC-CMs from the PEV groups showed a clear ability to enhance cell migration compared to ADSC-CM from the control group; this effect was achieved in a PEV concentration-dependent manner (Fig. 6B–E). We also investigated the effects of ADSC-CMs from all the groups on HUVEC tube formation with a Matrigel model and found that the ADSC-CM from the PEV groups demonstrated greater capacities to promote tube formation than that from the control group (Fig. 6F, G).

### Preconditioning with PEVs in vitro enhances the vasculogenic potential of ADSCs in vivo

**Fig. 7:**
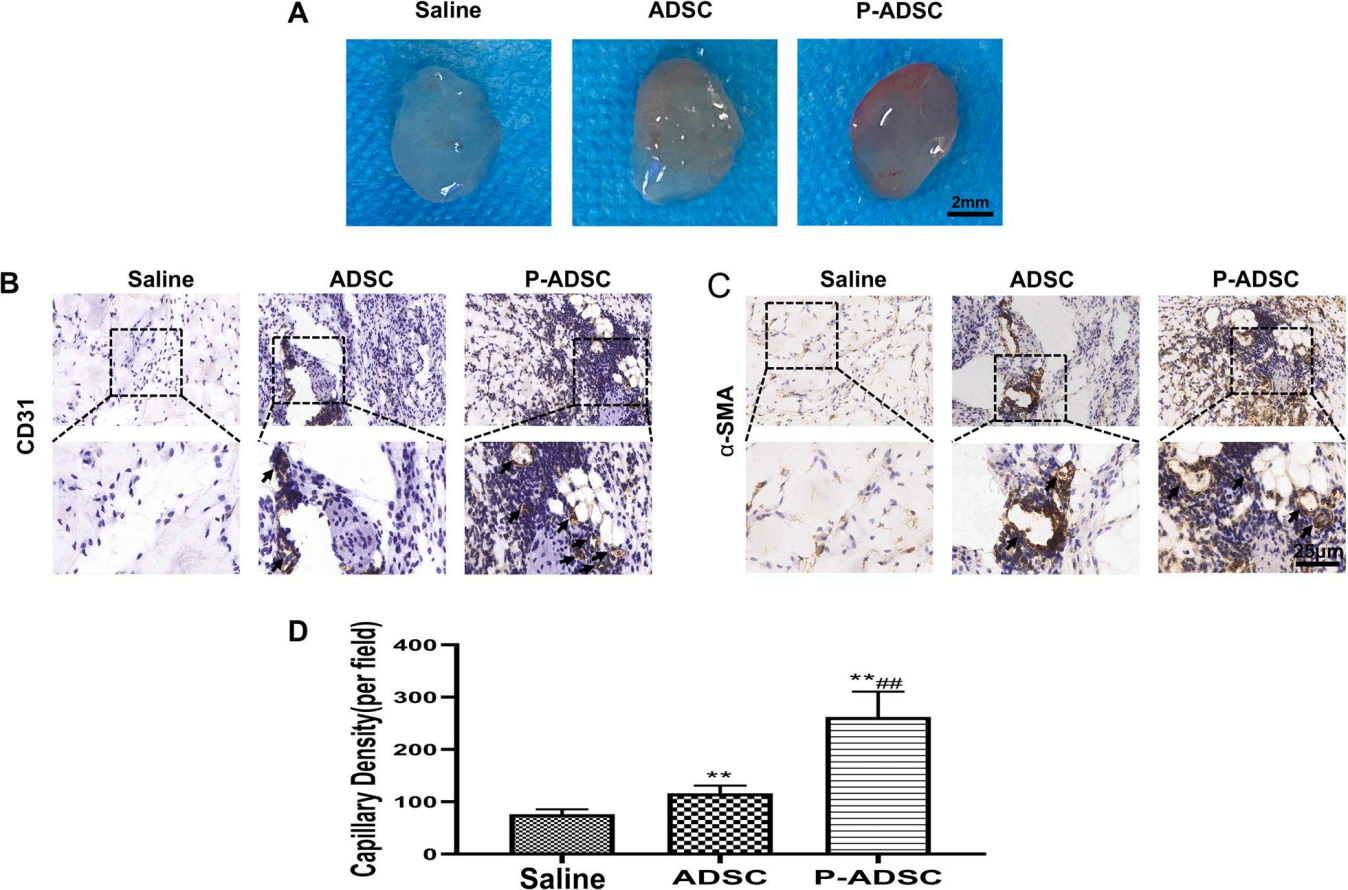
Matrigel plug assay showed proangiogenic properties of PBS, ADSC and P-ADSC in vivo. **A** Matrigel plugs derived from different groups. Matrigel mixed with PBS, ADSC and P-ADSC was subcutaneously injected into the abdomen of mouse. The plugs were harvested at 14 days after implantation. **B**, **C** CD31 and α-SMA immunostaining of Matrigel plug sections and positive staining showed in brown. P-ADSC group showed more blood vessels formation than PBS and ADSC groups; α-SMA, alpha-smooth muscle actin. **D** Statistical analysis of vessels number in Matrigel plugs. *p < 0.05, **p < 0.01 versus saline, #p < 0.05, ##p < 0.01 versus ADSC. P-ADSC PEV treated ADSCs

To study the effect of PEVs on the vasculogenic potential of ADSCs in vivo, a Matrigel plug assay was performed by subcutaneously injecting a Matrigel mixture, which included PBS, ADSCs and P-ADSCs, into mice. The gross view of the plugs harvested 14 days after implantation showed that reddish grafts were observed in the P-ADSC group, while nearly transparent grafts were observed in the PBS group (Fig. 7A). Capillary density was measured by immunohistochemical staining and showed that the numbers of CD31 + vascular structures (Fig. 7B) and α-SMA + cells (Fig. 7C) were significantly higher in the p-ADSC group than in the ADSC and control groups (Fig. 7D). In brief, these results suggested that preconditioning with PEVs in vitro could promote the angiogenesis potential of ADSCs in vivo**.**

### PEVs increase the therapeutic effects of ADSCs on hind limb ischaemia

**Fig. 8:**
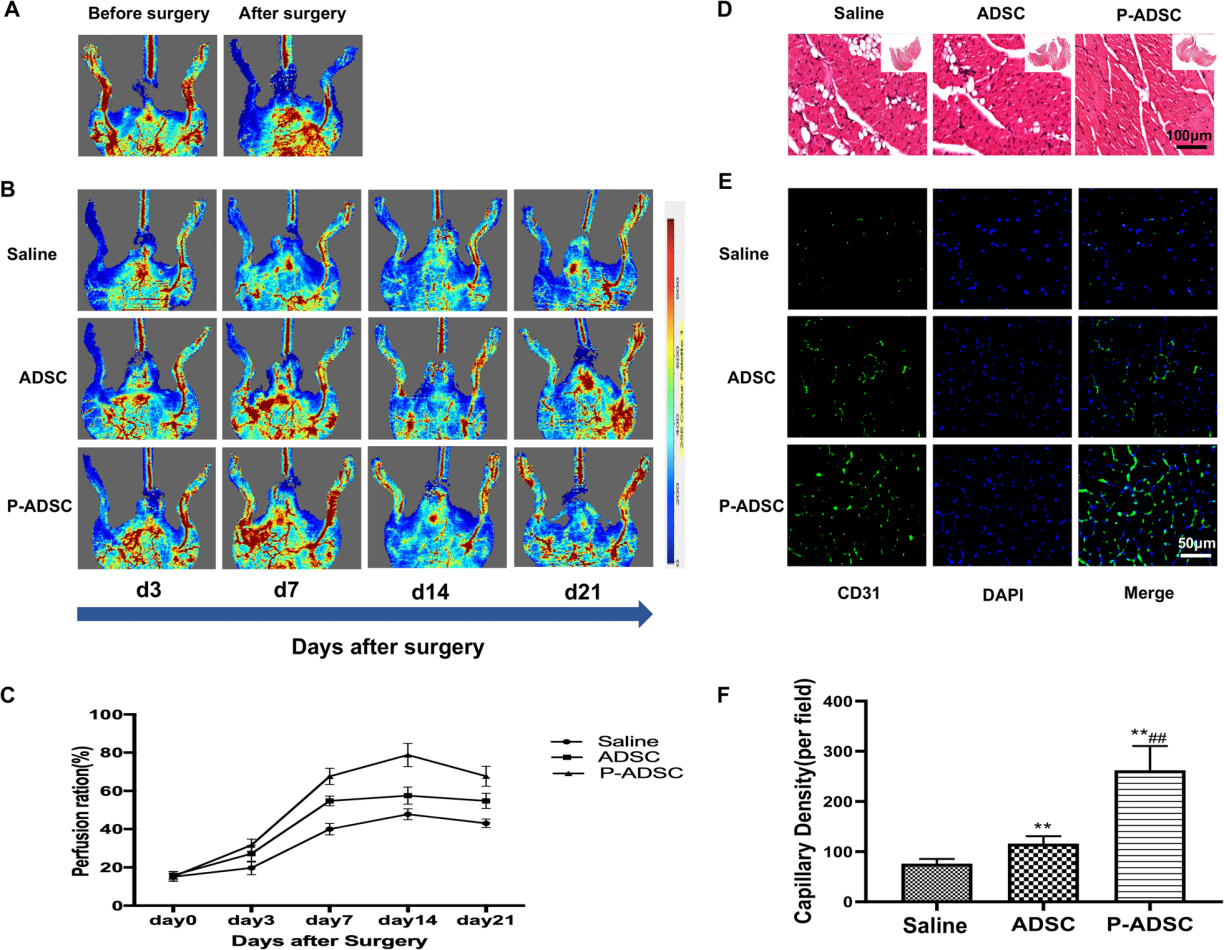
Mouse model showed P-ADSC improved perfusion recovery, attenuated degeneration and strengthened angiogenesis of ischemic hindlimb. **A** Representative laser perfusion Doppler image (LDPI) of mouse hindlimb before and after surgery. **B** Blood perfusion recovery in limb ischemia of each group at days 3, 7, 14, 21, post-surgery. **C** Quantitative analysis of blood recovery measured by LDPI. Blood recovery was represented using the ratio of ischemic hindlimb perfusion to contralateral hindlimb perfusion. **D **Haematoxylin–eosin (H&E) staining of gastrocnemius muscle of mouse ischemic hindlimb. **E**, **F** immunofluorescent staining of CD31 + capillaries in gastrocnemius muscles of ischemic tissue and quantification analysis. *p < 0.05, **p < 0.01 versus Saline, #p < 0.05, ##p < 0.01 versus ADSC. P-ADSC PEV-treated ADSCs

We investigated the angiogenic capacity of ADSCs preconditioned with PEVs in vivo using a hindlimb ischaemia mouse model. ADSCs cultured with PBS or PEVs (1 × 10^6^ cells, 100 µl; n = 10) were intramuscularly injected into ischaemic hindlimbs after ischaemia induction. Blood reperfusion recovered significantly better in the P-ADSC group than in the ADSC group (Fig. 8B, C), and nearly no blood reperfusion was observed in the PBS group at days 3, 7, 14 and 21. These findings suggested that PEVs could effectively amplify the therapeutic blood reperfusion induced by ADSC transplantation in vivo. In addition, we observed that the P-ADSC group showed lower degree vacuolar degeneration in muscle bundles than the other groups (Fig. 8D).

We further examined the neovascularization in the ischaemic hindlimbs of each group through histological analysis. The immunohistochemical results revealed that the capillary density of the ischaemic tissues in the PEV group was significantly increased compared with that in the other groups (Fig. 8E, F).

Therefore, PEVs could significantly enhance ADSC-induced angiogenesis in ischaemic tissues in vivo, exhibiting great potential in the promotion of stem cell therapy for treating CLI patients.

## Discussion

Over the past decade, therapeutic stem cell transplantation has attracted extensive attention in the treatment of ischaemic diseases [24, 25]. ADSCs are promising cell candidates because of their ease of isolation, abundance and strong proliferation ability compared with those of other cells, such as BM-derived stem cells [4]; in addition, the beneficial effects of ADSCs have been investigated in multiple animal models [26–28]. Paracrine secretion and cell migration are thought to be important mechanisms underlying the proangiogenic capacity of ADSCs in vivo [27, 29, 30]. However, these mechanisms are effective only when sufficient numbers of transplanted cells accumulate in the ischaemic region. Therefore, increasing the antiapoptotic activity and proliferation of ADSCs is also critical for producing therapeutic benefits [9].

PEVs are bioactive nanofragments released from platelets that contain abundant genetic material and proteins and can participate in many biological and pathological processes via intracellular communication; the processes in which PEVs participate include angiogenesis, inflammation and immunoregulation. A growing body of evidence suggests that PEVs can serve as transcellular delivery systems and regulate various functional features of target cells by delivering bioactive molecules. Studies have shown that PEVs can enhance the proangiogenic potential of EOCs by improving paracrine secretion and recruitment [31–33]. Here, we demonstrated, for the first time, that thrombin-/collagen-induced PEVs amplify the key functions of ADSCs by promoting ADSC proliferation, migration, antiapoptotic activity and paracrine activity, leading to improved angiogenic and vasculogenic performance in vivo.

PEVs can interact with recipient cells by binding, membrane assimilation and internalization [20, 33, 34]. In the present study, confocal laser microscopy provided evidence for the incorporation of CM-Dil-labelled PEVs by eGFP-ADSCs under different PEV concentrations and different time points, which indicated that PEVs could be internalized by ADSCs in a concentration- and time-dependent manner. This phenomenon is consistent with the concept of microparticles as “cargo”, which was mentioned above.

Migration is a major mechanism that facilitates the navigation of implanted cells from their initial position to sites of injury or inflammation [35]. Local transplantation circumvents the need for migration through the vascular system, so nonsystemic homing is based on migration through tissue in spatial proximity to the injury [35]. In the current study, we demonstrated that P-ADSCs exhibited increased migratory capacity, which might markedly drive ADSCs to migrate to the ischaemic border zone and exert their therapeutic effect.

A low grafting rate and limited paracrine capability have always been two limitations to stem cell-based therapy, so improving the survival rate and growth factor secretion of implanted cells is vital processes [36]. Furthermore, self-renewal and antiapoptotic capacities are essential for increasing the retention of stem cells implanted into ischaemic regions [37, 38]. In the current study, the CCK-8 assay revealed that PEVs could significantly promote ADSC proliferation, and 20 µg/ml was proved to be the optimal PEV concentration. Moreover, PEVs could enhance the antiapoptotic ability of ADSCs in the context of hypoxic environmental stimulation by CoCl_2**.**_These results indicated that P-ADSCs have enhanced viability and stronger anti-apoptotic abilities, which are essential for maintaining sufficient numbers of implanted ADSCs in the extreme environment created by ischaemic tissues. To explore the underlying mechanism by which PEVs affect ADSCs, we measured the expression of some classical pathway components and found that signalling downstream of PEVs was related to increases in pSrc, pFAK, pPLCγ1, pErk1/2 and pAkt, which are implicated in cell survival, migration and proliferation [39–42]. Based on these results, we hypothesized that PEVs may first phosphorylate FAK, PLCγ1 and Src after internalized by ADSCs, and then activate the downstream MAPK/Erk1/2 and PI3K/Akt pathways. However, the specific molecular interactions are still unclear and need to be further verified.

It is known that the secretion of proangiogenic cytokines by ADSCs is a potential mechanism of neovascularization [43]. In the present study, the results revealed significant upregulation of various angiogenic growth factors, including VEGF, HGF, PGF, TGF-β and Ang1, as measured by RT-PCR and Western blotting. This might suggest that PEVs may stimulate greater secretion of these factors and that a combination of these growth factors probably synergistically promotes angiogenesis and thus generates stable functional vessels. Because EC proliferation, migration and tubulization are the key steps in the process of angiogenesis [44] and cytokines secreted by ADSCs in vitro are present in CM [45], we explored the effect of PEVs on ADSC proangiogenic ability by investigating the biological effect of ADSC-CM on HUVECs in a coculture system. Consistent with the RT-PCR and Western blot results, the CM from P-ADSCs significantly increased the proangiogenic capacity of ADSCs in vitro. The dose-dependent effect of P-ADSC-CM on enhancing the proliferation, migration and tube formation abilities of HUVECs might be attributed to effects on the growth factors mentioned above and their cross-talk with other growth factors. All these results proved that PEVs, especially at a concentration of 20 µg/ml, could effectively improve the proangiogenic function of ADSCs by increasing angiogenic cytokine release, thus verifying the importance of PEVs in the treatment of chronic ischaemic patients.

The in vivo data demonstrated that P-ADSCs significantly enhanced the therapeutic angiogenic efficiency, as shown by the more extensive vessel structure in the Matrigel plugs and the higher capillary density in the mouse ischaemic hindlimbs. It is certain that in vivo angiogenesis represents a complex event that depends on the activation and interaction of a wide variety of molecules to initiate neovascularization and remodelling, which may account for the early vascular structure formation observed in the Matrigel plugs. Furthermore, the evident improvement in blood perfusion recovery observed in the mouse with hindlimb ischaemia at day 21 after P-ADSC transplantation should be attributed to synergistic effects of the PEVs on ADSCs, including the increased migration, proliferation and paracrine proangiogenic effects described above. The promotion of migration and proliferation capacities guarantees that sufficient numbers of implanted cells move from the injection site to the target region. At the ischaemic site, stronger antiapoptotic and proliferative abilities are associated with greater ADSC retention, and improved paracrine secretion can play a role. These results indicate that preconditioning with PEVs is a potential novel method for using ADSC-based therapy.

Together, these results provide strong evidence that PEV preconditioning serves as a prospective method for increasing the effect of stem cell-based therapy.

For now, no research has concentrated on the role of PEVs in the biological functions of ADSCs, which is vital for enhancing their clinical practice in patients with ischaemic disease. This study provides proof that PEVs can significantly improve the therapeutic efficiency of ADSCs. There are two limitations in the present study. The first limitation is the stem cell candidate we chose. We chose human-derived ADSCs to treat hindlimb ischaemia in mice via xenotransplantation. However, a number of studies have shown that mesenchymal stem cells have low immunogenicity, which allows them to survive in xenotransplantation and play their role in promoting regeneration. At present, the intracellular mechanisms have only been superficially explored, and further research is needed to understand the direct mechanism by which PEVs act on ADSCs.

## Conclusion

Our research proved that PEVs can effectively enhance ADSC viability, migration, and proangiogenic potential both in vitro and in vivo, leading to a better prognosis in an ischaemic model. Therefore, PEV-treated ADSCs can be used as an essential therapeutic choice for patients with disabling ischaemia.

## Data Availability

The datasets used and/or analysed during the current study are available from the corresponding author on reasonable request.

## Abbreviations

ADSC: Adipose-derived stem cell
PEVs: Platelet-derived macrovesicles
P-ADSC: PEV-treated ADSC
HUVEC: Human umbilical vein endothelial cells
CM: Conditioned medium
PRP: Platelet-rich plasma
VEGF: Vascular endothelial growth factor
HGF: Hepatocyte growth factor
PDGF: Platelet-derived growth factor
FGF: Fibroblast growth factor
PGF: Placental growth factor
eGFP: Enhanced green fluorescence protein
